# Acute increases in brain-derived neurotrophic factor following high or moderate-intensity exercise is accompanied with better cognition performance in obese adults

**DOI:** 10.1038/s41598-020-70326-1

**Published:** 2020-08-10

**Authors:** Daniela Sayuri Inoue, Paula Alves Monteiro, José Gerosa-Neto, Priscilla Rodrigues Santana, Fernando Pierin Peres, Kate M. Edwards, Fabio Santos Lira

**Affiliations:** 1 Exercise and Immunometabolism Research Group, Post-Graduation Program in Movement Sciences, Department of Physical Education, School of Technology and Sciences, State University (UNESP), Presidente Prudente, São Paulo Brazil; 2 Psychology Department, Universidade São Judas Tadeu and Fundação Santo André, São Paulo, Brazil; 3 Medicine Department, Universidade Do Oeste Paulista, São Paulo, Brazil; 4 Charles Perkins Centre, Faculty of Health Sciences, The University of Sydney, Sydney, NSW Australia

**Keywords:** Biochemistry, Immunology, Neuroscience, Physiology, Diseases, Endocrinology, Neurology

## Abstract

The purpose of this study was to test if different intensities of aerobic exercise could influence abdominal fat, isoforms of BDNF and executive function. Twenty obese men (30.0 ± 5.4 years old; 34.4 ± 3.5 kg/m^2^) were randomized to moderate-intensity continuous training (MICT, n = 10) and high-intensity intermittent training (HIIT, n = 10) three times a week for 6 weeks, with isoenergetic energetic expenditure for each exercise session (~ 300 kcal) between conditions. Abdominal fat was assessed pre- and post-intervention; executive function (Coding subtest from BETA-III non-verbal intelligence test and Stroop Color and Word Test), concentrations of mBDNF and proBDNF were assessed in response to acute exercise pre- and post-intervention. Abdominal fat did not change in either group. There was a significant increase in mBDNF immediately after acute exercise in both groups before and after intervention. proBDNF did not present changes acutely nor after 6 weeks. Executive function presented a main effect of time at pre- and post-intervention time-points Stroop Word and Stroop Color and Coding subtest presented improved performance from pre- to post-acute exercise session, in both groups. In conclusion, executive function improvements and acute exercise session-induced increases in mBDNF concentration were found from pre- to post-exercise intervention similarly between MICT and HIIT in obese men.

## Introduction

There is evidence of an association between augmented abdominal adiposity and lower cognitive function, and it seems that one link between these two parameters is through immunometabolic alterations provoked by pro-inflammatory cytokines, such as interleukin-6 (IL-6), released from adipose tissue^1–3^. Aspects of cognition, such as executive function, which includes working memory, inhibitory control and cognitive flexibility^2, 4, 5^, can be affected by the size of visceral fat^1^ and metabolic dysfunction^3, 6^, which are very common conditions in subjects with obesity. In this context, both abdominal fat and cognitive impairments seem to be associated with lower concentrations of brain-derived neurotrophic factor (BDNF)^2, 7^, an important pleiotropic protein directly related to neuron health and brain function^4^.

As a neurotrophin, there are different isoforms of BDNF, such as mature BDNF (mBDNF) and pro-BDNF (proBDNF). mBDNF corresponds to major part of total BDNF, activates tropomyosin-related kinase B receptor (TrkB) and culminates in signaling pathways involved with survival, plasticity and neurons functions^4^. proBDNF bind to p75 neurotrophin receptor (p75NTR) associated with sortilin complex which triggers signaling pathway of apoptosis, atrophy of axons and leads to a decrease of hippocampal density^8^ and neurodegeneration. These isoforms of BDNF seem to be related to nervous system function, including cognition. However, the majority of studies have not evaluated mBDNF and proBDNF separately, making it difficult to understand their real role. Given the association between obesity and cognitive impairment, it is especially important to evaluate BDNF isoforms in this population, and to evaluate the effects of strategies designed to minimize impairments on executive function from abdominal fat.

Exercise training is an important strategy in this context^9, 10^. Meta-analysis of Keating et al*.*^11^ and Wewege et al*.*^12^ confirm aerobic training performed at moderate- or high-intensity are able to reduce body fat. These two types of exercise intensity have been widely investigated, however, experiments evaluating obesity, cognition and BDNF all together, are still scarce. A meta-analysis of Chang et al.^13^ involving healthy subjects found acute exercise improves cognitive performance and it could be related to different factors, including increments of BDNF concentrations. Moreover, according to this meta-analysis, the intensity of acute exercise is considered as moderator variable, since high-intensity produced better cognitive results than moderate-intensity. However, the authors suggest more studies are needed to clarify the mechanisms that might explain these outcomes.

Brunelli et al.^14^ published one of the first studies verifying the impact of intensities of aerobic exercise on isoforms of BDNF in healthy and moderately trained men. They found PBMC intracellular content of proBDNF significantly elevated from baseline at 30 min and 60 min after the end of maximal incremental cycling, while a trial performed at anaerobic threshold, showed proBDNF elevated above baseline only at 60 min post-exercise. On the other hand, PBMC intracellular content of mBDNF increased significantly at 30 and 60 min post-exercise performed at intensity of anaerobic threshold, while after the maximal incremental cycling test, there was a significant increase immediately post-exercise followed by a reduction during recovery to levels below baseline at 60 min post-exercise. A similar exercise protocol was conducted by Piepmeier et al.^15^ with implementation of memory assessment, which investigated the responses of isoforms of BDNF according to intensity of exercise and their impact on cognitive variable in adult males. They found increases in mBDNF concentrations only in high-intensity exercise session, and no changes in proBDNF.

However, although such studies bring relevant results about impact of exercise on adiposity, cognition and isoforms of BDNF, the absence of workload equalization to compare these two types of intensity means interpretation must be cautious. Energy expenditure of exercise (EE/session) is a variable less explored when compared with intensity and volume of training. However, EE/session should be considered since its parameter is related to immunometabolic management^16^ including concentrations of BDNF^17^. The using of EE/session could be efficient for workload equalization when intending to compare different intensities of exercise, especially in weight loss programs^11, 18^, making the results more reliable.

Thus, the purpose of the present study was to test if different intensities of aerobic exercise could influence abdominal fat, concentrations of BDNF isoforms and executive function after 6 weeks of aerobic exercise training program with the same energy expenditure for each session. We expected high-intensity aerobic training to give greater changes in abdominal fat, executive function and isoforms of BDNF outcomes.

## Materials and methods

### Subjects

This study is part of a main project entitled “The impact of two Models of Aerobic Training on Cognitive Function, Morphological and Systemic Immunometabolic Changes of Young People with Obesity” (RBR-7pdc8y). Participants were recruited via advertisements (social networks, printed posters, and radio/television) and selected according to inclusion (healthy young adult male, 18–36 years old; sedentary ≤ 1 day/week of structure physical activity; BMI 28–35 kg/m^2^, outlined by WHO et al*.*^19^; medical consent) and exclusion criteria (any cardiovascular disease; diabetes; orthopedic/neurological limitation; severe depression according Beck Depression Inventory; smoking; use of medications or drugs; and abuse of alcohol) verified during the very first interview. Since our protocol is a short intervention (6 weeks) and there would not be possible to control changes of specific sex hormones as estrogen which has a strong and direct association with BDNF (Scharman and MacLusky 2007), only males were included to make the sample more homogeneous. The study was developed in accordance with the Helsinki Declaration and approved by the Research Ethics Committee from Faculty of Science and Technology from Universidade Estadual Paulista (FCT-UNESP) of Presidente Prudente city (reference number: CAAE 46948215.8.0000.5402).

Since visceral fat is considered as a cornerstone of immunometabolic alterations presented in obesity and seems to be related with cognitive impairments (as described in “Introduction” section), the power of analysis was calculated based on this variable. A minimal sample size of n = 14 (7 participants per group) was determined by a power calculation (G*Power version 3.1.9.2; Heinrich Heine University Düsseldorf, Düsseldorf, Germany) using an [alpha] of 5%, a 1-[beta] of 80%, and an effect size of 0.886 based on visceral fat values from the pilot study performed previously.

### Study design

**Figure 1:**
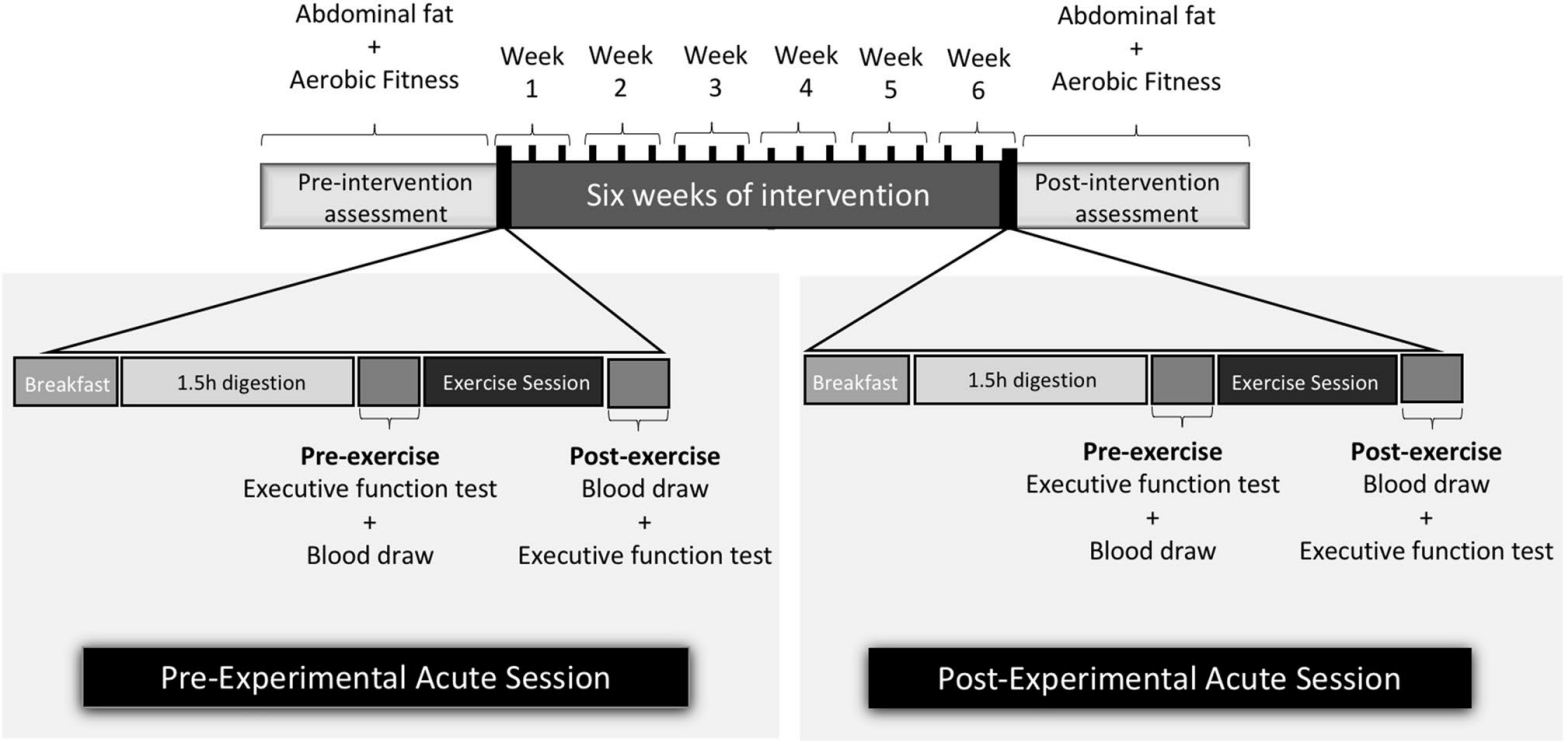
Study design.

One hundred seventy-two people were interviewed and fifty-seven men were able to participate and provide the written informed consent, then, they were submitted to cardiologic assessment. Eligible participants had their visceral fat and aerobic fitness assessed to stratified randomization in three different groups: moderate-intensity continuous training (MICT), high-intensity intermittent training (HIIT) and Control. However, because the purpose of this present study was to assess different intensities of acute exercise on isoforms of BDNF and executive function, we did not evaluate these parameters in a Control group, therefore, they were not included here. Each MICT or HIIT participant performed 2 weeks of familiarization and then, they started 6 weeks of exercise training (period of intervention). The first (at Week 1) and the last (at Week 6) day of exercise training consisted of experimental acute sessions, with executive function assessments and blood draws. After 6 weeks of intervention, all variables were re-assessed (Fig. 1). It was required that participants maintained their habitual food intake and avoid of other types of physical training across the period of intervention. For data analysis, all participants who had a minimal frequency of 75% during intervention were included.

### Aerobic fitness assessment

Participants performed an incremental speed test on treadmill (1% inclined) until voluntary exhaustion. Each stage consisted of one minute. The initial speed was 5.5 km/h with 0.5 km/h increments per stage until voluntary exhaustion. Rate of oxygen consumption (VO_2_) was measured during the test (Model Quark PFT Ergo—COSMED—Rome). It was considered VO_2_max when there was stabilization of the maximum oxygen consumption between the last stages and VO_2_peak when there was not. The maximal aerobic velocity (MAV) corresponded to the speed reached in VO_2_max or VO_2_peak. At the end of each stage the participants reported the rate perceive exertion (RPE)^20^.

### Energy expenditure estimation and equalization

The estimation of energy expenditure was assessed and applied to make the workload equalization between MICT and HIIT groups and allow an appropriate comparison of results. The protocol to determine the energy expenditure of the exercise session (EE/session) is detailed in Gerosa-Neto et al.^18^. Participants of both groups had no statistical difference in EE/session, and considered as isoenergetic aerobic training.

### Exercise training programs

MICT and HIIT groups performed 6 weeks (three sessions per week) of supervised running on treadmill. HIIT consisted of general 5-min warm-up on the treadmill at 5 km/h and following of total 10 bouts of intermittent running on the treadmill (10 × 1:1—one minute at 100% of MAV—interspersed with one minute of passive recovery). In the MICT group the warm-up was the same as the HIIT group, but the training session consisted of supervised walking/running on treadmill continuously at a velocity corresponding to 65% MAV. The training volume (total minutes per session, ≈ 40 min) was determined according to the corresponding total EE/session of a single previous session of HIIT performed by each MICT participant. Thus, there was a matched EE between MICT and HIIT protocols.

### Abdominal fat assessment

The evaluation of visceral fat was performed by an ultrasound method in an institution specialized in image diagnosis. The ultrasound determination of subcutaneous fat was previously defined as the distance between the skin and the outer face of the rectus abdominis muscle, and visceral fat was defined as the distance between the internal face of the same muscle and the anterior wall of the aorta. The points of the interruption to define the visceral fat by methodological parameters were based on previous descriptions by Ribeiro-Filho et al.^21^. The pre- and post-assessment were performed by the same radiologist.

### Executive function assessment

Executive function was evaluated through the Coding Subtests from BETA-III non-verbal intelligence test^22^, and Stroop Color and Word Test^23^. They were applied pre-exercise (immediately before blood draw) and post-exercise (immediately after blood draw) in both experimental acute session—the first (at Week 1) and last (at Week 6) day of exercise training (Fig. 1). The Coding Subtests from BETA-III non-verbal intelligence test^22^, is based on transcribing codes (symbols) according to the corresponding number. The tests were performed by paper version in limit time of two minutes. The number of correct answers defined the score. The Stroop Color and Word Test^23^ applied is a paper version and analogous to Stroop Neuropsychological Screening test^24^. This test was composed for two tasks in incongruent condition: (1) to name 112 color words printed in different ink colors (Stroop Word); (2) to name the ink colors of 112 color words (Stroop Color). There is a limit time of 2 min per task, however participants needed to read aloud and as soon as possible all 112 items/task. The time of execution (in seconds), was applied as score. There was a practice cognitive session prior to baseline data collection, which occurred with a minimal interval of 1 week.

### Experimental acute sessions

Participants were instructed to refrain from performing physical exercises (exercise or unusual activities) for 72 h and 12 h of fasting before each experimental acute session (at the first and last day of intervention training—on Week 1 and Week 6, respectively). Upon arrival at the test site, a standardized breakfast (25% of daily energy need according body composition—distributed in 52% of carbohydrate, 35% of lipids and 13% of protein) consisting of toasts, yogurt and cottage cheese was offered. After 1.5 h of interval (digestion), participants were submitted to executive function tests, blood draw and then, exercise session (see details in “Exercise Training Programs” section). Immediately after performing exercise, there was a blood draw and executive function test, respectively (Fig. 1).

### Blood sample and analysis

Eight milliliters (ml) of blood sample were collected in tubes containing separator gel (BD SST II Advance Becton Dickinson, BD), immediately before (pre-exercise) and immediately after exercise session (post-exercise) in both experimental acute session (at Week 1 and Week 6; Fig. 1). The blood sample were centrifuged at 3,000 rpm for 15 min at 4 °C. Serum was then stored in microcentrifuge tube and stored at -20° C for further analysis. IL-6 (R&D Systems Quantikine ELISA kits, USA; Sensitivity 0.7 pg/ml; Assay Range 3.1–300 pg/ml) and Neurotrophins (Adipo Bioscience, USA) were analyzed by enzyme-linked immunosorbent assay (ELISA), according manufacturer’s instruction. For BDNF (cod. SK00752-01; sensitivity: 5–8 pg/ml; range 23–1500 pg/ml) analysis, serum samples were diluted 40-fold in dilution buffer while for proBDNF (SK00752-09; sensitivity 0.25 ng/ml; range 0.78–25 ng/ml) analysis run undiluted according to the manufacturer's suggestion.

### Statistical analyses

A three-way mixed analysis of variance (ANOVA) employing the repeated factors of “intervention time” (2 levels: Pre-intervention, Post-intervention) and “exercise time” (2 levels: Pre-exercise, Post-exercise), along with the no-repeated factor of “training type” (2 levels: HIIT, MICT) was used to analyze the dependent variables of BDNF, proBDNF, Stroop Word and Stroop Color Test and Coding Subtests (score). In addition, a two-way mixed ANOVA with the repeated factor of “intervention time” and the non-repeated factor of “training type” was used to analyze the dependent variables of BMI, total body mass, abdominal fat, IL-6, aerobic fitness (VO_2_max and MAV) and EE/session. Tukey’s post-hoc was utilized when a significant difference was found. In both two-way and three-way ANOVA the Mauchley sphericity test was tested, and when appropriate Greenhouse–Geisser test was applied. Statistical analysis was conducted utilizing IBM SPSS version 22 for Windows. The partial eta square (η_p_^2^) values were calculated by Jasp version 0.12.2. In all analysis alpha was set priori to *p* = 0.05.

## Results

A total of 20 participants (30.0 ± 5.4 years old; 34.4 ± 3.5 kg/m^2^) could complete and perform the final evaluations. Table 1 presents the results of morphological and physiological parameters at pre- and post-intervention.

**Table 1:** Morphological and physiological parameters pre- and post-intervention.

Variable	Group	Week 1	Week 6	Effect	F	*p*	η_p_^2^
Body mass (kg)	MICT (n = 10)	108.2 ± 12.1	107.9 ± 12.5	Period	1.358	0.259	0.070
	HIIT (n = 10)	106.8 ± 9.1	106.3 ± 9.8	Group	0.092	0.765	0.005
				Period*Group	0.054	0.819	0.003
BMI (kg/m^2^)	MICT (n = 10)	34.6 ± 3.7	34.5 ± 3.9	Period	1.395	0.253	0.072
	HIIT (n = 10)	34.1 ± 3.6	34.0 ± 3.7	Group	0.071	0.793	0.004
				Period*Group	0.066	0.800	0.004
Visceral fat (cm)	MICT (n = 10)	7.1 ± 1.7	7.1 ± 1.9	Period	0.371	0.550	0.020
	HIIT (n = 10)	5.9 ± 1.4	5.6 ± 1.0	Group	4.049	0.059	0.184
				Period*Group	0.371	0.550	0.020
Subcutaneous Fat (cm)	MICT (n = 10)	2.6 ± 0.7	2.9 ± 1.2	Period	0.005	0.943	(–)
	HIIT (n = 10)	2.7 ± 0.6	2.5 ± 0.7	Group	0.314	0.582	0.017
				Period*Group	4.429	0.050	0.197
IL-6 (pg/mL)	MICT (n = 10)	1.80 ± 0.75	1.62 ± 0.52	Period	0.102	0.753	0.006
	HIIT (n = 10)	1.38 ± 0.43	1.47 ± 0.67	Group	1.360	0.259	0.016
				Period*Group	1.077	0.313	0.056
MAV (km/h)^#^	MICT (n = 10)	9.2 ± 0.7	9.7 ± 0.7*	**Period**	**23.915**	**< 0.001**	**0.555**
	HIIT (n = 10)	10.0 ± 8,75	10.7 ± 1.2*	**Group**	**5.129**	**0.036**	**0.222**
				Period*Group	1.161	0.295	0.061
EE/session (kcal)	MICT (n = 10)	292.6 ± 33.6	311.6 ± 39.7*	**Period**	**6.543**	**0.022**	**0.298**
	HIIT (n = 07)	290.9 ± 36.2	322.2 ± 39.7*	Group	0.082	0.779	0.005
				Period*Group	0.387	0.543	0.025

Values expressed by mean ± standard deviation.

*MAV* Maximal aerobic velocity, *EE/session* energy expenditure per exercise session.

^#^Difference between group. *Different from pre-intervention to the same group (*p* < 0.001). (–) no detected by Jasp program. [Bold] (*p* < 00.05).

**Figure 2:**
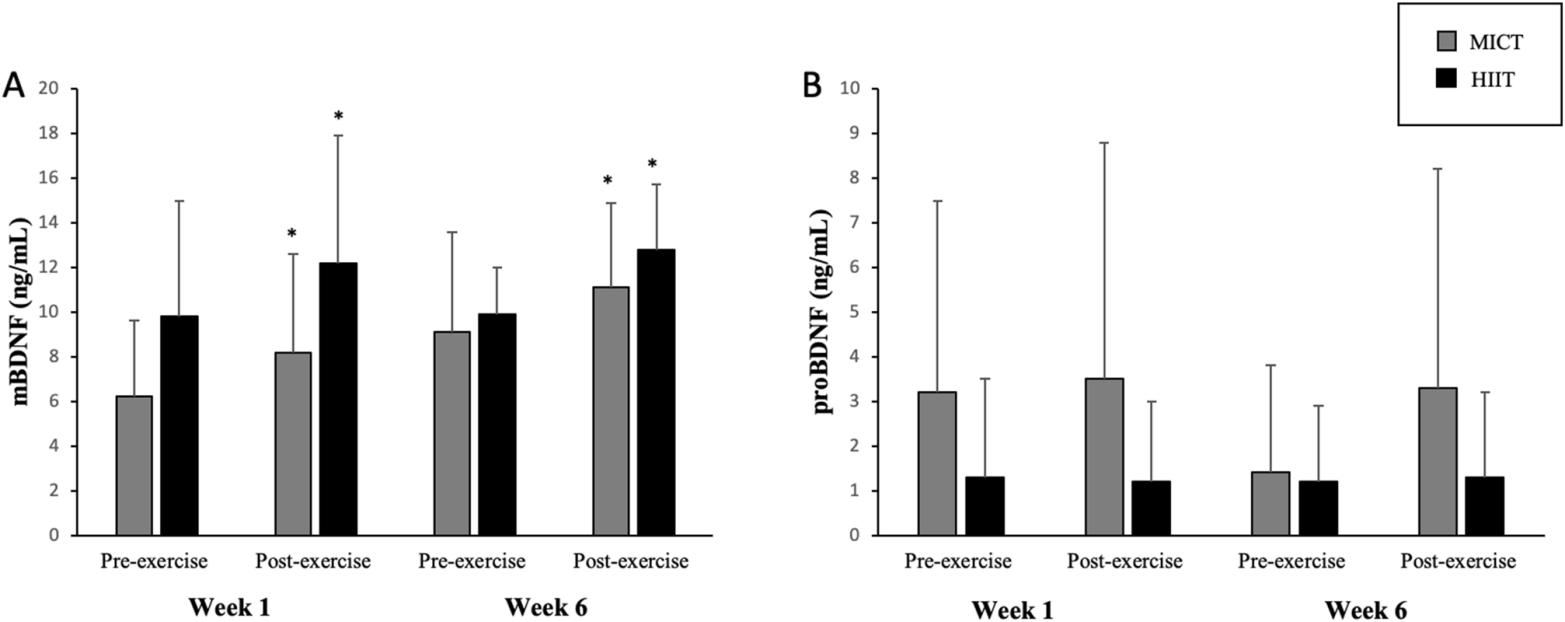
mBDNF (**A**) and proBDNF (**B**) values in acute exercise session in Week 1 and Week 6. *Significant difference from pre-exercise to same moment. The values are presented in mean (bar) and standard deviation (error bar).

One participant (HIIT group) was excluded from mBDNF analysis since its sample referent of Week 6 could not be measured. There was a main effect of time for mBDNF (F_(1,17)_ = 40.557, *p* < 0.001, η^2^ = 0.693) with lower values in pre- than post-exercise (experimental acute exercise session) in both Week 1 and Week 6 in both MICT and HIIT groups. For concentrations of proBDNF analysis, three participants had an undetectable value (HIIT = 1; MICT = 2). There was no significant difference in any condition (Fig. 2).

Table 2 present the statistical results of executive function. There were occasional missing data in Stroop Color (HIIT = 1; MICT = 1) and Stroop Word and Coding Subtest (HIIT = 2; MICT = 1), due to technical errors.

**Table 2:** Executive function.

			Pre-exercise Mean ± SD	Post-exercise Mean ± SD	Acute effects	F	*p*	η^2^	Chronic effects	F	*p*	η_p_^2^
Stroop word (s)	MICT (n = 09)	Week 1	75.6 ± 16.5	63.0 ± 13.2^a^	**Session**	**16.031**	**0.001**	**0.517**	**Period**	**16.997**	**0.01**	**0.531**
		Week 6	66.7 ± 16.1^1^	58.9 ± 9.4^a1^	Session*Group	0.713	0.412	0.045	Group	0.096	0.761	0.006
	HIIT (n = 08)	Week 1	74.3 ± 20.5	64.5 ± 14.3^a^	Session*Period	1.554	0.232	0.094	Period*Group	0.896	0.359	0.056
		Week 6	60.8 ± 11.6^1^	57.3 ± 10.3^a1^					Period*Sesssion*Group	0.028	0.870	0.002
Stroop color (s)	MICT (n = 09)	Week 1	107.2 ± 15.4	102.4 ± 18.3^a^	**Period**	**39.835**	**0.001**	**0.713**	**Period**	**28.573**	**0.001**	**0.641**
		Week 6	99.9 ± 17.4^1^	92.0 ± 15.1^a1^	Session*Group	0.227	0.640	0.014	Group	0.032	0.861	0.002
	HIIT (n = 09)	Week 1	108.9 ± 13.7	103.4 ± 16.2^a^	Session*Period	0.223	0.640	0.014	Period*Group	0.001	0.974	(–)
		Week 6	99.9 ± 15.3^1^	94.4 ± 17.4^a1^					Period*Sesssion*Group	0.223	0.643	0.014
Coding subtest (score)	MICT (n = 08)	Week 1	70.8 ± 14.9	83.6 ± 23.6^a^	**Period**	**38.587**	**0.001**	**0.720**	**Period**	**21.844**	**0.001**	**0.593**
		Week 6	73.9 ± 14.4^1^	91.6 ± 21.8^a1^	Session*Group	0.028	0.868	0.002	Group	0.500	0.490	0.032
	HIIT (n = 09)	Week 1	63.4 ± 8.0	78.9 ± 17.8^a^	Session*Period	2.803	0.115	0.157	Period*Group	0.577	0.459	0.037
		Week 6	70.4 ± 6.1^1^	87.3 ± 12.3^a1^					Period*Sesssion*Group	0.826	0.378	0.052

Values expressed by mean ± standard deviation.

^a^Different from pre-exercise to same period and group (*p* < 0.001).

^1^Different from Week 1 period to same moment and group (*p* < 0.001). (–) no detected by Jasp program. [Bold] (*p* <  0.05).

## Discussion

To our knowledge, this study was the first to investigate if different intensities (moderate- and high-intensity) of aerobic exercise could influence abdominal fat, cognitive function and isoforms of BDNF (mBDNF and proBDNF) after 6 weeks of aerobic training program with same energy expenditure in each exercise session. The results of this study did not confirm our initial hypothesis which was high-intensity aerobic training would give greater changes in those variables.

Regarding aerobic fitness, MAV and EE/session increased in both groups from pre- to post-intervention, evidencing that there was an improvement in physical performance. This could be explained by a neuromuscular adaptation, since repeated practice of exercise is able to increase the control of movement and muscle recruitment patterns^25^.

No statistical change was found for abdominal fat in either MICT or HIIT. In a meta-analysis by Keating et al.^11^, when MICT and HIIT are matched for EE/workload, similar small body fat reductions were expected even without any difference in total body mass. Here, weight and fat losses were not observed MICT and HIIT after 6 weeks. It has previously been shown that for weight loss, dietary intervention combined with exercise is required^12^. As we examined exercise effects alone this finding is therefore not unexpected.

Abdominal fat (visceral and subcutaneous fat) did not present significant reductions in adipose tissue size (cm), however there was a trend for HIIT to decrease subcutaneous fat (*p* = 0.050, η^2^ = 0.197) more than MICT. This finding is of note given that higher abdominal fat has shown be related to lower cognitive function^2, 6^ thought to be because the excess of abdominal fat favors a pro-inflammatory environment that influences cognition negatively. Although subcutaneous fat has a predominantly protective metabolic characteristic^26, 27^, it has been demonstrated that this part of abdominal fat tissue, in its deepest layer (below Scarpa fascia), has the capacity for greater expansion and appears to be strongly related to metabolic disorder similarly to visceral adipose tissue^27^. Thus, reduction of this parameter would be extremely relevant, and these trends toward change were observed for the HIIT group and not MICT. IL-6 did not change from pre- to post-intervention in either group, which might be expected given that no significant differences were found in visceral fat, a main resource of inflammation in obesity^1–3^. In addition, Batacan et al.^28^ argue the effect of HIIT on cytokines, such as IL-6, is still not clear, especially because the number of studies is small and there is high variability among protocols (shorter and longer).

Regarding BDNF isoforms, the concentrations of proBDNF in the present study did not change acutely nor after 6 weeks, as in agreement with the study of Piepmeier et al.^15^, while mBDNF increased in both experimental acute exercise sessions, independent of group. These results are relevant because they show the concentration of proBDNF did not change in the same way of mBDNF after a single bout of exercise or short-term training. Interestingly, although the baseline concentration of proBDNF remained the same from pre- to post-intervention, it did not negatively impact the cognitive performance of the participants, who showed improved performance after 6 weeks of training. On the other hand, it is important to note that three participants in this study had undetectable concentrations as happened in the study of Yoshida et al.^29^. Therefore, further studies, including improvement in sensitivity of techniques, are necessary to clarify impact of exercise on proBDNF function and its mechanisms.

Regarding specifically acute exercise responses, concentrations of mBDNF increased immediately after acute exercise, in agreement with previous results from other studies with different populations^30–32^. We did not observe any difference between acute responses to HIIT and MICT, unlike the study by Piepmeier et al.^15^, in which they found increases of this neurotrophin only in vigorous intensity. Our results indicate EE/session might be an important variable rather than intensity per se. This acute elevation of mBDNF may be related to several associated factors. First, exercise increases IGF-1 concentrations and this hormone seems to be closely related to the production of BDNF by neurons^33, 34^. Second, contraction of skeletal muscle during exercise increases the production and action of transcription factors, such as PGC-1α, Errα and Fndc5, which appear to play a role in regulating BDNF transcription^35, 36^.

Although baseline mBDNF concentrations did not change from baseline to post 6 weeks of intervention here, our results are in agreement with the results of Griffin et al.^32^ who also did not detect changes after 5 weeks of intervention in sedentary young non-obese adults, although only BDNF and not isoforms were measured. In contrast, studies with interventions longer than 12 weeks have shown an increase in basal concentrations of BDNF^7, 35–37^. This suggests that more studies regarding changes in resting BDNF isoforms are required to determine the effects of training and specifically duration of intervention.

In the present study, executive function increased from pre- to post-intervention in both MICT and HIIT groups, and increased acutely in response to exercise similarly between groups. Increases were found in performance of each of; Coding Subtest from the BETA-III non-verbal intelligence test, Stroop Word and Stroop Color tests after the acute exercise session as well as baseline values after 6 weeks of intervention. Our results are similar to the study of Griffin et al.^32^ that found improvements in Stroop Word and Color test after 5 weeks of moderate-intensity training in sedentary young non-obese adults. Griffin et al*.*^32^ also found in sedentary non-obese adults an increase of BDNF concentration concomitant to cognitive performance in acute exercise, suggesting a relationship between both variables, but we were not able to replicate this finding. Hashimoto et al.^38^ suggest there is no relation between BDNF and executive function improvements after a bout high-intensity interval exercise (HIIE), and such responses in acute cognition might come from energy substrate resource, such as lactate. In addition, although the present study did not include executive function assessment after exercise recovery, others have found a better sustained Stroop task performance in HIIE than moderate-intensity continuous exercise after 30 min passive recovery^39^. Although Tsukamoto et al.^39^ did not measure concentrations of BDNF, other studies support the suggestion that this neurotrophin starts to return to baseline values immediately after exercise ending^31, 40^, therefore, suggesting the sustained acute executive function performance is independent of acute concentrations of BDNF.

This study has some limitations. First, cleavage proteases, TrkB and p75NTR were not assessed. To know if functions of these variables are influenced by exercise could give a better understanding of BDNF isoforms action in obese population. Second, our period of intervention was only 6 weeks, limiting clarifying whether a longer-term intervention would be effective in increasing baseline mBDNF concentrations. Another limitation is there is no non-exercising control group to compare effects of training over the intervention.

In conclusion, executive function improvements and acute exercise session-induced increases in mBDNF concentration were found from pre- to post-exercise intervention similarly between MICT and HIIT in obese men, even without abdominal fat changes. Our results suggest energy expenditure as a fundamental variable which needs to be considered in future studies that investigate the impact of intensity of exercise.
